# Remarks on Dr. Buxton's Treatment of Cough

**Published:** 1813-07

**Authors:** 


*To the Editors of the Medical and Physical Journal.*

"Dedi cor meum ut scirem prudentiam atque doctrinam erroresque et stultitiam; et agnovi quod in his esset labor et afflictio spiritus."—*Eccl.*

GENTLEMEN,

IT is amusing to see the various ways by which practitioners in medicine endeavor to procure themselves notoriety. It is, also, a pleasing rather than a hurtful vanity to their brethren, for it enables them to discriminate characters divested of obsequiousness, grimace, and professional humbug, and as it were to dissect them by the blaze of their own productions.

The trick of writing a book on a disease to be cured by medicine to be found in any modern pharmacopœia, is now proved to be very stale and unprofitable; nay often injurious to ill-founded reputation, as appears by many living examples: therefore it was necessary, to succeed, to discover something new and strange.

Ever since Dr. Buxton's visit to Germany, and falling in love with one of their stoves, we have had little books and little essays, on winter cough, regulated temperature, and German stoves.

Dr. Buxton procured a room, in some lane at Horsleydown, (I presume for the benefit of carbonic acid gas also) about the dimensions of 15 feet by 15, and heated by a German stove, which projected 5½ feet into the room. The temperature required was 65°, from which it seldom varied considerably. Into this room he introduced several patients suffering from catarrh, &c. in order to put his opinion to the test of experiment concerning the benefits to be derived from a regulated temperature.

The first case, Osborn, a young man, aged 21, had, on the 2d of January, been ill six weeks from catarrh, owing to exposure to wet, and coughed frequently, and expectorated freely, without any pain of chest. Had continued to work, but felt weak.

"He began to take antimonial wine thrice a-day, and, on the 6th, had a blister applied to the sternum, and dressed with savine ointment, and tincture of squills added to the antimonial wine.—On the 8th was still better, and permitted to walk out, during which it rained, and he got triflingly wet! Complaints much aggravated.—10th. Blister repeated, with savine ointment and ant. wine.—12th. Better in every respect.—15th. Blistered part continues to discharge.—17th. Rather better.—18th. Blister scarcely draws. Spermaceti ointment.—19th. The same. Antimonial wine and tincture of squills. Blister and savine ointment.—22d. Some aggravation of symptoms since last report; are now abated. Blister does not discharge much.—24th. On the whole rather better.—27. Some aggravation of symptoms, since last report, are now abated.—29th. Nearly the same.—30th. Osborn *says* that his cough is now very much better than when he was admitted, particularly during the day: expectoration and breathing also considerably relieved, but not to so great a degree as either of the other symptoms. He is not so well as he was about twelve days ago, but is not at all worse than he has been for some days past."—Discharged.

The result of the first case, so far from proving the advantage of regulated temperature, appears directly the opposite, and, I may venture to add, was highly injurious to the patient, as shall be proved.

What can be judged from a history wherein the state of the tongue, of the appetite, of the bowels, of the skin, of the pulse, is never mentioned, nor during the whole disease, excepting that once the pulse was said to be *now* rather quick! And what is not less deserving of censure, the medicine given is mentioned without any dose being specified. Now, though Dr. B.'s practice, in that respect, may be known at the London Hospital, it cannot even be guessed at by those who do not belong to that institution. The names of the remedies mentioned might be, and are, employed in very different doses, according to the intention of the prescriber, and with very different results.

Another omission, of no small importance, is the diet which was permitted in this conservatory, the regulation of which,

 probably, probably, did no less towards the cure than even the regulated temperature. But I deny that any cure was performed, or even the slightest benefit produced.

To say nothing of the impropriety of permitting a man, who had been cooped up in a room at 65° during six days in January, even to walk out, was not his getting wet relinquishing all principle in treating the disease? and who knows that the man did not aggravate the evils by drinking something to keep the *wet* from his stomach!

Twice during his confinement did Osborn suffer from an increase of the symptoms without any evident cause. But is Dr. B. ignorant that in pulmonic disease, even where the lungs are irremediably diseased, the cessation and aggravation of cough, &c. in the same room, confined to the same bed, and with a temperature not less accurately kept, and *without any evident cause*, is one of the most familiar occurrences to be met with?

Three blisters, and those kept discharging, with the use of antimonial wine, and tincture of squills, judiciously managed, are remedies of no small power; yet here, because as Lord Bacon says "Not idols of the den," they are accounted nothing. I have no doubt, that, upon a similar principle, had Radford, of Cheapside, sold the patient a fleecy hosiery *night-cap*, he would have attributed, and with no less impropriety, all the benefit to his night-cap, which Dr. Buxton does to his regulated temperature.

But how lamentable, that, after four weeks, the report of the 30th should prove that Osborn was little relieved, and not so well as he had been twelve days before, in which state he was discharged! Yet here we are requested to admire the benefit arising from regulated temperature and German stoves!

I shall now prove, that, instead of an advantage, he derived a positive injury from the treatment.

A tradesman (in a business, I believe, liable to great variations of temperature) is taken, in the month of January, and shut up for four weeks, at nearly one temperature, during which he is principally trusted to the benefit proposed to result therefrom; though, at the same time, using some remedies of considerable efficacy. On the 1st of February he is not cured, indeed scarcely better in any way, yet he is then discharged to meet with all the variations of temperature he had formerly suffered, and doubly aggravated by being infinitely more liable to feel their bad effects; and now sufficient to render permanent what was before remediable!

diable! therefore he may justly be said to have suffered considerable injury from the plan of treatment.

It may be added, too, that whether this was a summer or a winter cough, an autumn or a spring one, it was evidently not accompanied by any alarming symptoms. Now experience teaches, and from no small share of practice, even amongst the lower orders, and with all the excesses and improprieties of their ignorance and unrestrained conduct during treatment, that no instance of cough, so trifling, has ever been half so little benefited in the same space of time, though it might reasonably be doubted that some of the medicine was not taken.

The treatment, undoubtedly, was very different from the trifling here enumerated, but how much better for the individual who could receive double benefit, without quitting the bosom of his family for a temporary jail, or being rendered doubly susceptible of disease!

The remaining eight cases were so nearly similar, that I shall not occupy time, or the Journal, in detailing them, but only notice some particular differences in their history and treatment.

Case 2d.—Is a better described case, and a more severe one. Treatment antimonial wine and blisters, with an aloetic pill for a few nights. Was a patient above five weeks. The 14th and 16th of February reports are occasional pains between the shoulders; chest pains him a little; fits of dyspnœa just as a week ago; expectoration deep colored, but not red, and trifling in quantity; pain in left side of the head; small of back very weak; cough farther diminished. 19th. Was not within! 20th. Discharged!

A patient, under the benefit of regulated temperature, suffered to be out, on the 19th of February, in our climate.

The word discharged, on the following day, having no accompaniment, we must conclude he received no further advantage than last reported.

Case 3d.—Richard Hughes, aged 20, ill eight months, had been under Dr. B.'s care since September, was admitted January 20th. "Coughs much; breath very short; raises much mucus, which he expectorates easily; perspires both in the day and night; is very weak; tongue clean; bowels regular. Antimonial wine, perpetual blisters, and sulphuric acid. At the end of four weeks, wheezing nearly as before; breath still better; strength further increased; does not perspire so much. Discharged."

It would be a bold assertion to say that he was materially better, on reading the whole case, for although some symptoms toms had abated, the lungs still remained oppressed; and it would be still bolder to assert that the medicines had no share in producing the trifling advantage.

Case 4th.—"John Corney, aged 17, ill six months. Is delicate and narrow chested; cough; wheezing; occasional vomiting from coughing; expectoration moderate. Is weak, emaciated, and pulse quick! Been a patient since September, since which his complaints have been better, but is now weaker, and thinner." Has used laxatives, antimony, squills, perpetual blisters, and sulph. acid. "Repeat the sulph. acid and perpetual blister, and laxative pill occasionally." After several aggravations and remissions of symptoms, at the end of the month the report was, "Coughed much yesterday and last night, very little to-day; expectoration last night increased, but not this morning; throat not at all stopped; appetite nearly as it was. Discharged."

From some of the reports, it is to be presumed he went out weaker than on his entrance, and certainly not more advanced towards a cure!

Case 5th.—"George Bell, aged 55, ill four months, but for ten years had been asthmatic during winter, but in summer quite free from disease.

"Em. Ol. Ant."

What that is we are unable even to guess, and being unwilling to attribute the brevity of the prescription to ignorance or conceit, beg Dr. B. will explain his hieroglyphics, and the dose.

On the fourth day the report says "mouth feels sore since yesterday," which occasioned us to look again at the Em. Ol. Ant. to see if this soreness could be produced by the medicine, as no clue is given to discover its cause. If I was disappointed in that, I was again puzzled at the new prescription it had occasioned, which seems even to have startled the Editor.

Aq. Menth. pip. ℥i, ter die.

"No one," says Dr. Buxton, "can possibly conceive that these articles were of material consequence to his relief." As a proof of the propriety of this remark, his mouth on the 14th was only "nearly well;" on the 20th he was discharged by his own desire, with giddiness in his head, &c. and with his breathing only affected in the evening. Is this a wonderful alleviation in asthma, though his temperature had remained unchanged?

Case 6th.—William Tonks, aged 61, ill three months; asthmatic. In summer is very well! Was ill six weeks last winter!—February 17th. A perpetual blister, and Em. Ol. Ant.

Ant. 17th. Another blister. Liq. Ant. Tart. P. coloc. i. o. n.—March 5th. So ill in the night that he required to be visited by the apothecary, who ordered his bowels, which were confined, to be opened, and so relieved him. Another blister.—9th. Another blister.—19th. Another blister.—21st. Discharged; *says he* is better in every respect; had been in five weeks.

The 7th, 8th, and 9th cases present nearly similar, though on the whole less favorable, results; and the practice was distinguished by its usual conciseness.

The hint, that two persons to whom this room was recommended, and refused its benefits, are since dead, and that some of those whose cases have been detailed, might, probably, not now have been alive! is rather too abstruse a calculation to establish such data upon. It would be more instructive to know that all these cases were cured, and alive, and healthy, on the 1st of June, than to hear conjectures of what might and what might not be.

After the failure of the cow-house scheme, and others of a similar nature, we are sorry to see a regular-bred physician imitating the Grahams, and Solomons, and Brodums of the day, by inventing the paltry name of winter cough, to alarm the credulous, to confide in his *peculiar practice* of curing it.

A cough may proceed from various causes, none of which are peculiar to winter or summer; and it is a doctrine, not discovered by Dr. B. but as old as Hippocrates, that variations of temperature are hurtful, and to be avoided in all pulmonic diseases; though it was reserved for Dr. Buxton to attempt *to cure them* by so doing. But even this doctrine is controverted by great experience, and not delusive but real success. Sydenham's cure for consumption was riding on horseback, and Dr. Rush mentions, in his essays, where fatigue, constant exposure to vicissitudes, &c. cured several hopeless cases. It would be well that such plans were again resorted to, instead of the enervating, consuming, and miserable one of sitting by a German stove with all the horrors of a jail.

We would also recommend Dr. B. to revise his medicinal plan of treating catarrhal and consumptive diseases, as some better acquaintance with remedies more appropriate than antimonial wine and tincture of squills may be acquired, and with a success which will far outstrip all the pretended virtues of a cruel and comfortless confinement, which can only perpetuate the ravages of the disease.

That such may be the effects of our remarks is the sole intention of our venturing to make them, and with a wish to to deter others from so fruitless and inefficacious a remedy as a warm room and a tea spoonful of mint water three times a-day, while a vulture lies within devouring them!

I am, Gentlemen,

*London*, Your obedient Servant,

*June* 4, 1813. A PRACTICAL PHYSICIAN.*

* The author of this paper will observe that we have omitted some passages which he might think witty, but which, in our opinion, were more sarcastic than is consistent with professional courtesy, especially when directed by an anonymous writer against the practice of a physician of established reputation.—EDITORS.

